# Effects of Arbuscular Mycorrhizal Fungi on the Growth and Competitive Ability of *Solanum rostratum*, an Invasive Plant

**DOI:** 10.3390/plants15162517

**Published:** 2026-08-20

**Authors:** Zheng Lyu, Yangcheng Shi, Siying Meng, Pengbo Yin, Siqi Zhu, Zhenwen Xu, Guijun Wang, Helong Bai

**Affiliations:** 1 College of Geography, Changchun Normal University, Changchun 130032, China; 2 College of Chemistry, Changchun Normal University, Changchun 130032, China

**Keywords:** *Solanum rostratum*, AM fungi, high-throughput sequencing, compartmented pot, interspecific competition

## Abstract

*Solanum rostratum* is an invasive plant species that poses a serious threat to native ecosystems. In this study, we investigated *S. rostratum* populations in saline-alkali regions of western Jilin Province to characterize root-associated arbuscular mycorrhizal (AM) fungal diversity and evaluate the effects of AM fungi on plant growth and competitive ability. The results showed that *S. rostratum* roots were widely colonized by AM fungi, indicating abundant AM fungal resources in its rhizosphere. High-throughput sequencing identified 45 AM fungal species belonging to 13 genera, with *Glomus* as the dominant genus. A compartmented mesh pot experiment further demonstrated that AM fungal inoculation significantly enhanced the growth and photosynthetic performance of *S. rostratum*, particularly under saline-alkali soil conditions. When AM fungi were inoculated only in the compartment containing the native plant *Setaria viridis*, AM fungal colonization was also detected in *S. rostratum* roots, indicating hyphal connections between neighboring plants through the mesh barrier. Stable isotope analysis further revealed that AM fungi facilitated nitrogen acquisition by *S. rostratum* and increased ^15^N transfer from neighboring *S. viridis* under common mycorrhizal networks. These findings suggest that abundant AM fungal resources in the rhizosphere contribute to the growth advantage of *S. rostratum*. By enhancing nutrient acquisition and potentially facilitating nitrogen transfer through mycorrhizal networks, AM fungi may strengthen the competitive ability of this invasive plant under saline-alkali conditions.

## 1. Introduction

*Solanum rostratum*, also known as buffalobur, is an annual herbaceous plant native to North America and a member of the genus *Solanum* in the family Solanaceae. It is widely distributed across Europe, Africa, and Australia [1,2]. In China, *S. rostratum* was first discovered in Chaoyang, Liaoning Province, in 1981. Over time, this species has expanded into northern, northeastern, northwestern, and eastern China, and has recently become established in a wide range of natural habitats [3,4].

The plant is covered with prickly hairs throughout, which can harm livestock, reduce pasture quality, and lower wool production. Additionally, *S. rostratum* serves as a field host for the potato leafroll virus, posing a serious threat to local ecological environments [5,6]. *Setaria viridis* is an annual herbaceous plant belonging to the genus *Setaria* in the family Poaceae. It is native to temperate and warm temperate regions of Eurasia and is now widely distributed across temperate, warm temperate, and tropical regions worldwide [7]. During field surveys of *S. rostratum* populations in western Jilin Province, we observed that *S. viridis* was one of the most common associated plant species in *S. rostratum* habitats. Therefore, *S. viridis* was selected as the competitor species in this study.

Arbuscular mycorrhizal (AM) fungi are a group of fungi that form symbiotic structures with plant roots. In this mutualistic association, plants supply carbon sources and other nutrients to AM fungi, while AM fungi extend the root absorption area through their hyphal networks, facilitating the uptake of water and nutrients such as nitrogen, phosphorus, and potassium from the soil. Consequently, AM fungi enhance plant growth, productivity, and tolerance to environmental stress [8].

The role of AM fungi in biological invasions has received increasing attention in recent years [9]. Previous studies have demonstrated that AM fungi generally promote the growth and competitive ability of various invasive plant species [10]. Studies have shown that inoculation with AM fungi significantly enhances growth indicators such as biomass and plant height of the invasive plants *Wedelia trilobata* [11] and *Solidago canadensis* [12], while also significantly increasing their competitive effects, thereby facilitating invasion into newly colonized habitats.

In recent years, *S. rostratum* has proliferated in western Jilin Province, causing severe damage to the local ecological environment [13]. Although numerous studies have demonstrated that AM fungi can promote the growth, nutrient acquisition, and competitive ability of invasive plants [14], most of these studies have been conducted under non-stressful soil conditions, and the underlying mechanisms are mainly attributed to enhanced nutrient acquisition by host plants mediated by AM fungi [15]. However, it remains unclear whether AM fungi can still confer competitive advantages to invasive plants under saline-alkali stress conditions and whether such advantages are mediated by nutrient redistribution among plants through belowground mycorrhizal networks [16]. In saline-alkali ecosystems, resource limitation may further increase plant dependence on mycorrhizal symbiosis [17]; therefore, the mechanisms underlying AM fungal-mediated resource competition among plants require further investigation. Furthermore, current studies on the interactions between AM fungi and invasive plants have mainly focused on a limited number of representative invasive species [18], and systematic investigations into whether and how AM fungi influence the invasion process of *S. rostratum* remain lacking. Therefore, elucidating the effects of AM fungi on the growth and competitive ability of *S. rostratum* is of great ecological significance.

In this study, we investigated *S. rostratum* populations in saline-alkali habitats of western Jilin Province and combined field surveys, high-throughput sequencing, and compartmented mesh pot experiments to characterize the composition of root-associated AM fungal communities and determine their effects on plant growth, competitive ability, and nitrogen acquisition. Based on previous studies and the characteristics of saline-alkali habitats, we hypothesized that AM fungi would exert stronger positive effects on *S. rostratum* under saline-alkali conditions than under nutrient-rich soil conditions and that AM fungi may enhance its competitive advantage by increasing nitrogen acquisition through belowground mycorrhizal networks.

## 2. Materials and Methods

### 2.1. Field Sampling

**Figure 1:**
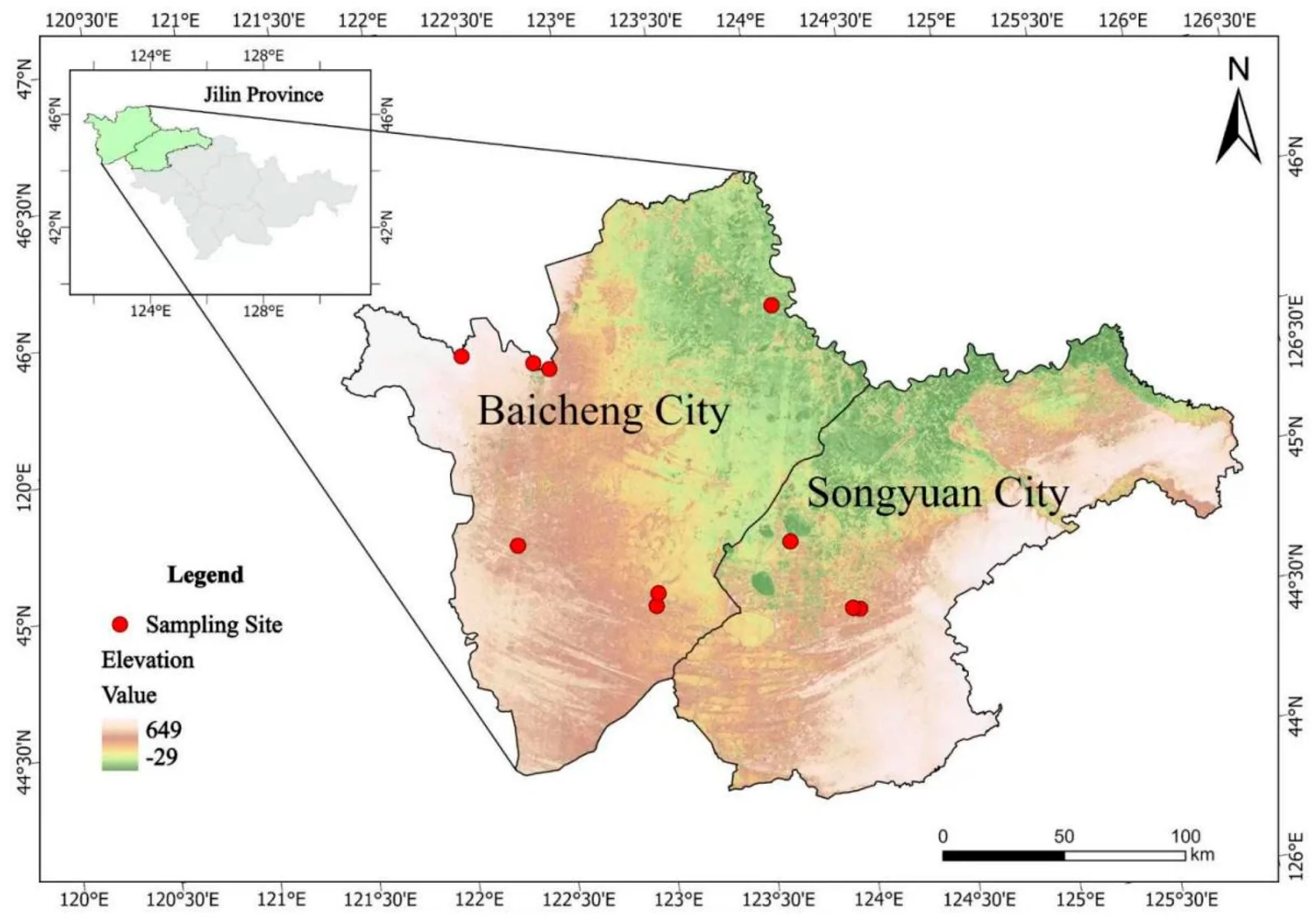
Geographical location of the sampling sites.

The western region of Jilin Province is located in the southwestern part of the Songnen Plain and has a temperate continental monsoon climate, with an annual mean temperature of 4–6 °C and annual precipitation of 400–500 mm [19]. Precipitation is concentrated in summer, while spring droughts, sandstorms, and cold, dry winters are common. The topography is dominated by alluvial plains, and the land is characterized by severe salinization. The main soil types are light chernozem, meadow soil, and saline-alkali soil. After a field survey of the *S. rostratum*-growing areas in western Jilin Province, ten representative sites were selected for sampling in Songyuan City and Baicheng City (Figure 1). At each site, three 1 m × 1 m quadrats were established, and five *S. rostratum* plants were randomly selected within each quadrat. Entire plants were collected and placed separately into sealed bags. Rhizosphere soil of *S. rostratum* was collected, mixed, and preserved at low temperature for high-throughput sequencing.

### 2.2. Compartmented Mesh Pot Experiment

To investigate whether the effects of AM fungi on *S. rostratum* differed between soil substrates, peat soil and saline-alkali soil collected from Qian’an County, Songyuan City, western Jilin Province, were selected as experimental substrates. Peat soil and saline-alkali soil were passed through an 18-mesh sieve to remove stones, plant residues, and other impurities. River sand was first sieved through an 18-mesh sieve to remove larger particles and impurities, followed by repeated washing with distilled water until the washing solution became clear and free of visible contaminants. A nutrient-rich soil substrate was prepared by mixing peat soil and river sand at a volume ratio of 2:1, while a saline-alkali soil substrate was prepared by mixing saline-alkali soil and river sand at the same ratio. The two soil substrates were transferred into high-temperature sterilization bags and sterilized by autoclaving at 121 °C and 0.1 MPa for 120 min.

The AM fungal inocula used in this study were purchased from the Institute of Plant Nutrition and Resources Science, Beijing Academy of Agriculture and Forestry Sciences. The inocula consisted of *Funneliformis mosseae*, *Glomus versiforme*, and *Rhizophagus intraradices*. The original inocula were propagated using *Trifolium repens* as a host plant to obtain pure inocula containing individual AM fungal species. The spore densities of the propagated inocula were determined using a stereomicroscope, resulting in 997 spores per 50 g for *F. mosseae*, 869 spores per 50 g for *G. versiforme*, and 967 spores per 50 g for *R. intraradices*. Equal amounts (10 g each) of the three AM fungal inocula were mixed to generate a composite inoculum. The resulting 30 g mixed inoculum contained approximately 650 AM fungal spores.

**Figure 2:**
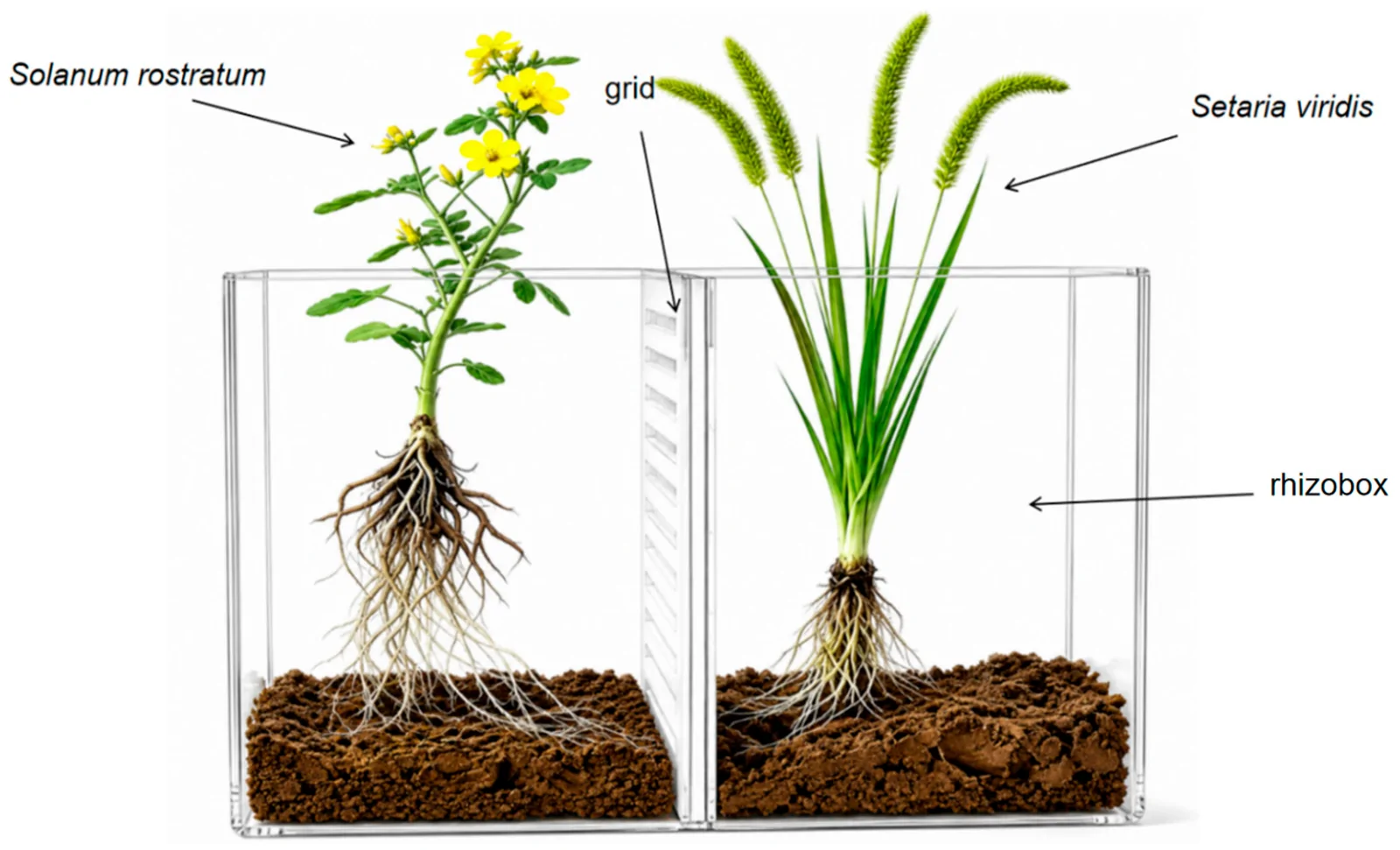
Schematic diagram of the compartmented mesh pot experiment.

Six treatments were established in the compartmented mesh pot experiment (three treatments for saline-alkali soil substrate and three for nutrient-rich soil substrate), with ten replicates per treatment (Table 1). The experiment used acrylic compartmented mesh pots with dimensions of 20 cm × 10 cm × 15 cm (length × width × height). Each pot was divided into two compartments by a perforated acrylic partition (2 mm thick). The partition featured uniformly distributed circular holes (5 mm in diameter) spaced 1 cm apart, forming an air gap to minimize nutrient transport via mass flow and diffusion. A 37.5 μm nylon mesh was embedded in the center of the partition, allowing the passage of AM fungal hyphae but excluding plant roots (Figure 2).

The experiment was conducted in June 2025. Seeds of *S. rostratum* and *S. viridis* were sterilized with 10% hydrogen peroxide solution for 10 min, rinsed with distilled water, and treated with gibberellic acid to break dormancy. After germination, the seeds were sown in seedling trays filled with peat soil (autoclaved). When the plants reached 30 days of growth (*S. rostratum* had developed four true leaves), healthy individuals of similar size were selected for the compartmented mesh pot experiment. For the AM fungal inoculation treatment, 30 g of AM fungal inoculum (containing *Funneliformis mosseae*, *Glomus versiforme*, and *Rhizophagus intraradices*, with approximately 650 spores) was added when the growth substrate reached three-quarters of the pot volume. Correspondingly, the control treatment received an equal amount of sterilized inoculum. Pots were watered every other day to maintain adequate soil moisture, and Hoagland’s nutrient solution was added every seven days. The experiment lasted for 60 days. During the experiment, the root boxes were regularly repositioned to minimize microclimatic variations among treatments. From June to September 2025, for a total of 12 weeks, the monthly average temperature was 21 °C.

Seven weeks after transplanting *S. rostratum* and *S. viridis* into the mesh pots, ^15^N labeling was conducted. The isotope used in this study was white crystalline K^15^NO_3_ (99 atom%^15^N). The isotope labeling was applied to the plants at a volume of 1 mL of 0.007 mol·L^−1^ K^15^NO_3_ per cm^3^ of soil, and labeling was performed exclusively on plants in Compartment I (Table 1). Sampling was conducted one week after labeling. The samples were air-dried and sent to Chengdu Baihui Biotechnology Co., Ltd. (Chengdu, China) for determination of ^15^N atom% excess. Three replicates were set for each treatment.

### 2.3. Identification of AM Fungi

Staining was performed using the method of Phillips and Hayman (1970). Root segments were examined under a microscope to assess AM fungal colonization. The colonization status of each root segment was recorded based on microscopic observations. The colonization frequency (F%), colonization intensity (M%), and arbuscule abundance (a%) of AM fungi in the rhizosphere of *S. rostratum* were calculated using MYCOCALC software. Samples were sent to Sangon Biotech (Shanghai, China) Co., Ltd. for high-throughput sequencing. The specific procedures were as follows: DNA was extracted from the samples using the E.Z.N.A.™ Mag-Bind Soil DNA Kit (Omega Bio-tek, Norcross, GA, USA), and PCR amplification was performed using the arbuscular mycorrhizal fungus SSU primers AMV4.5NF (AAGCTCGTAGTTGAATTTCG) and AMDGR (CCCAACTATCCCTATTAATCAT).

### 2.4. Plant Growth Attributes

Plant heights of *S. rostratum* and *S. viridis* were measured using a tape measure, while stem diameter and leaf thickness were measured using a vernier caliper. The number of leaves, flowers, and fruits was also recorded. After measurement, each plant was separated into flower/fruit, stem/leaf, and belowground parts. Samples were oven-dried at 105 °C for 30 min to inactivate enzymes, then dried at 75 °C to a constant weight. During the flowering and fruiting period, on a clear day around noon, leaf SPAD (Soil–Plant Analysis Development) values were determined using a portable chlorophyll meter (SPAD-502 Plus, Konica Minolta, Tokyo, Japan). Photosynthetic parameters—net photosynthetic rate (Pn), stomatal conductance (Gs), transpiration rate (Tr), and intercellular CO_2_ concentration (Ci)—were measured using a portable photosynthesis system (Li-6400, Li-Cor, Lincoln, NE, USA).

### 2.5. Analytical Analyses

The ^15^N atom% excess isotope was analyzed using isotope ratio mass spectrometry (IRMS). Organic carbon content in plant tissues was determined by the potassium dichromate oxidation-external heating method. Total phosphorus content was determined by the molybdenum-antimony colorimetric method after acid digestion. Total nitrogen content was determined using a Kjeldahl nitrogen analyzer.

### 2.6. Statistical Analysis

Data were recorded, organized, and calculated using Microsoft Office Excel 2021. Homogeneity of variance tests, general linear model (GLM), one-way analysis of variance (one-way ANOVA), Duncan’s multiple range test, and Pearson correlation analysis were performed using IBM SPSS Statistics 27. The GLM was used to evaluate the effects of soil substrate, AM fungi, and their interaction on the measured parameters. One-way ANOVA followed by Duncan’s multiple range test was only used to compare differences among the treatment combinations. Figures were created using Origin 2021 software.

## 3. Results

### 3.1. AM Fungi Colonization and Spore Density in the Rhizosphere of S. rostratum

AM fungi colonized the roots of *S. rostratum* at all ten sampling sites, with relatively high colonization frequencies ranging from 72.22% to 90.21% (Table 2), indicating that *S. rostratum* readily forms well-established symbiotic associations with AM fungi in western Jilin Province. Colonization intensities across the different sites ranged from 13.99% to 29.10%, with the highest value (29.10%) observed at site LYT and the lowest (13.99%) at site JCZ. Arbuscule abundances across the ten sites ranged from 8.36% to 19.65%; sites LYT, YLH, and EQS exhibited relatively high arbuscule abundances (19.65%, 17.87%, and 17.30%, respectively), while sites YST, XZC, and JCZ showed lower values (8.36%, 8.83%, and 9.40%, respectively). Furthermore, spore density differed significantly among sites (*p* < 0.05). The lowest spore density was recorded at site YLH, and the highest at site XZC, with a difference of 224.67 spores per 100 g of dry soil between the two.

### 3.2. AM Fungi Community Composition in the Rhizosphere of S. rostratum

High-throughput sequencing analysis was performed on rhizosphere soil samples of *S. rostratum* collected from ten sampling sites in western Jilin Province. A total of 2360 operational taxonomic units (OTUs) belonging to AM fungi were detected across all samples. After rigorous filtering of the raw sequencing data and precise annotation at the species level, a total of 45 AM fungal species were identified, belonging to 13 genera, 8 families, and 4 orders within the phylum Glomeromycota. The identified genera included *Archaeospora*, *Ambispora*, *Acaulospora*, *Diversispora*, *Scutellospora*, *Claroideoglomus*, *Dominikia*, *Funneliformis*, *Glomus*, *Rhizoglomus*, *Rhizophagus*, *Septoglomus*, and *Paraglomus* (Table 3).

### 3.3. AM Fungi Colonization Under Different Treatments

The compartmented mesh pot experiment (Table 4 and Table 5) showed that both *S. rostratum* and *S. viridis* in the inoculation treatments exhibited varying degrees of AM fungal colonization, whereas no colonization was observed in the non-inoculated treatments. Among the inoculated treatments, certain differences in colonization frequency were observed in *S. rostratum* under different treatment conditions. In treatments N2 and S2, where AM fungi were added only to the *S. viridis* side, *S. rostratum* roots were still colonized, with colonization frequencies of 61.84% and 47.78%, respectively. Regarding colonization intensity, *S. rostratum* in treatment S3 exhibited a colonization intensity of 32.79%, which was significantly higher than that in treatments N2 and S2 (*p* < 0.05). Arbuscule abundance results showed that *S. rostratum* in treatment S3 also had a significantly higher arbuscule abundance (9.82%) than treatments N2 and S2 (*p* < 0.05). In contrast, *S. viridis* showed overall lower AM fungal colonization frequencies, ranging from 44.45% to 68.51%, with colonization intensities between 8.31% and 16.46%, and generally low arbuscule abundances. These findings indicate that the colonization status of both *S. rostratum* and *S. viridis* was poorer under the saline-alkali soil substrates than nutrient-rich soil substrates, and that *S. rostratum* exhibited higher colonization levels than *S. viridis* overall.

### 3.4. Growth Responses of S. rostratum and S. viridis to AM Fungi Inoculation

**Figure 3:**
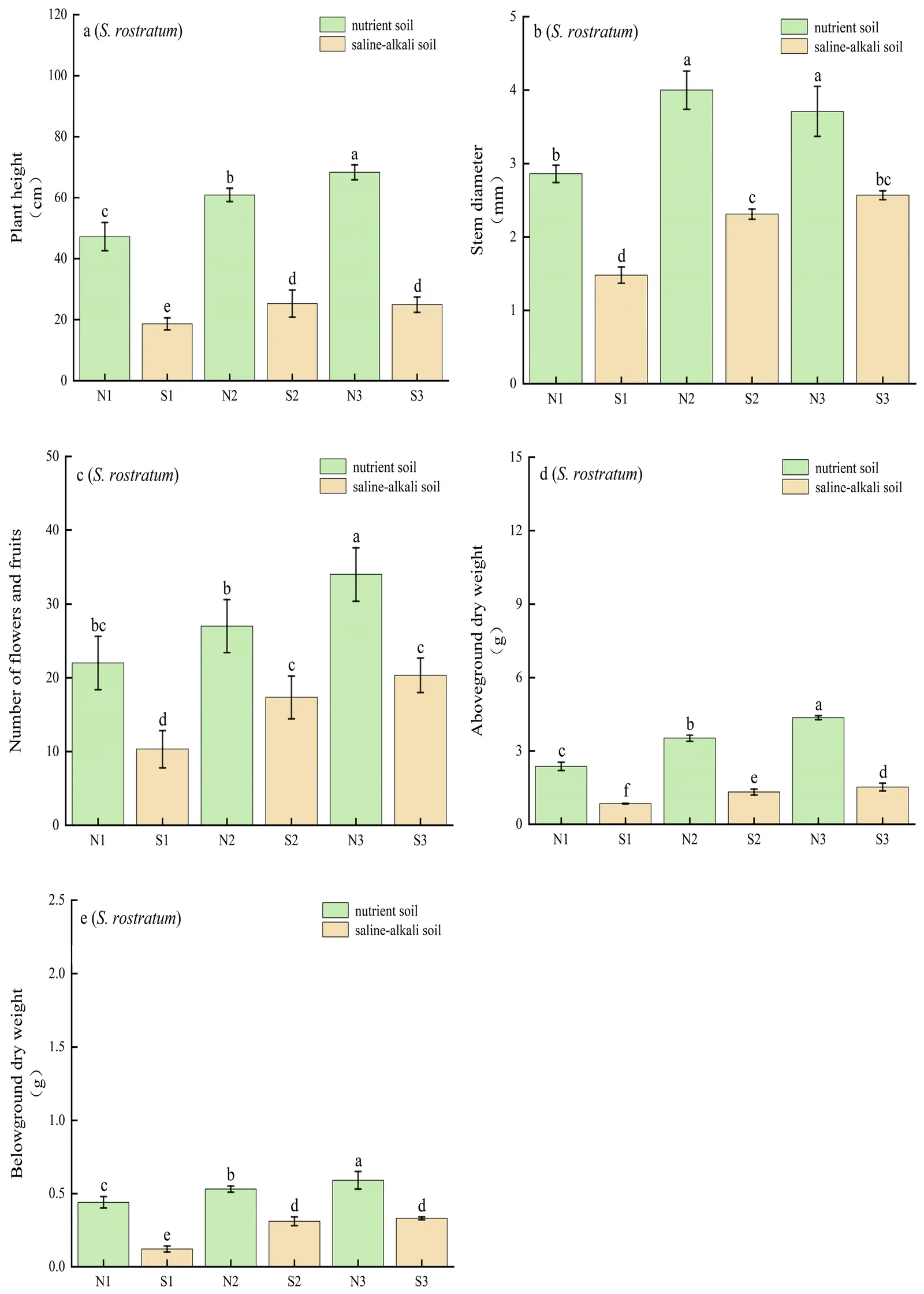
(**a**–**e**) Growth and biomass parameters of *S. rostratum.* Note: Different lowercase letters indicate significant differences among plants under different treatments (*p* < 0.05).

As shown in Figure 3, under nutrient-rich soil substrates, *S. rostratum* in treatments N2 and N3 exhibited overall significantly higher values than treatment N1 (*p* < 0.05). All indicators in treatment N3 were significantly higher than those in treatment N1 (*p* < 0.05), with stem diameter, number of flowers and fruits, and belowground dry weight increasing by 29.12%, 54.44%, and 34.09%, respectively. In treatment N2, stem diameter, number of flowers and fruits, and aboveground dry weight were also significantly higher than those in treatment N1 (*p* < 0.05). Plant height and aboveground dry weight in treatment N3 were significantly higher than those in treatment N2, with increases of 12.15% and 23.86%, respectively (*p* < 0.05). Compared with nutrient-rich soil substrate, the plant height, stem diameter, number of flowers and fruits, and aboveground and belowground dry weights of *S. rostratum* were significantly lower under the saline-alkali soil substrate (*p* < 0.05). However, all indicators in treatment S3 (with AM fungal inoculation) were significantly higher than those in treatment S1 (*p* < 0.05), with stem diameter, number of flowers and fruits, and belowground dry weight increasing by 73.65%, 96.81%, and 175%, respectively. Treatment S2 also showed significantly higher plant height, stem diameter, number of flowers and fruits, and aboveground and belowground dry weights than treatment S1 (*p* < 0.05), although these values remained substantially lower than those in the inoculated nutrient-rich soil treatments. Stem diameter in treatment S3 was significantly higher than that in treatment S2, with an increase of 11.26% (*p* < 0.05).

Under nutrient-rich soil substrates, stem diameter, aboveground dry weight, and belowground dry weight of *S. viridis* in treatment N2 were significantly lower than those in treatment N1, with reductions of 18.06%, 37.72%, and 28.64%, respectively (*p* < 0.05). In treatment N3, aboveground and belowground dry weights were significantly lower than those in treatment N1, with reductions of 35.68% and 22.54%, respectively (*p* < 0.05). Under the saline-alkali soil substrate, all indicators in treatments S3 and S2 were significantly higher than those in treatment S1 (*p* < 0.05). However, stem diameter, aboveground dry weight, and belowground dry weight in treatment S3 were significantly lower than those in treatment S2, with reductions of 20.12%, 26.88%, and 55.10%, respectively (*p* < 0.05).

**Figure 4:**
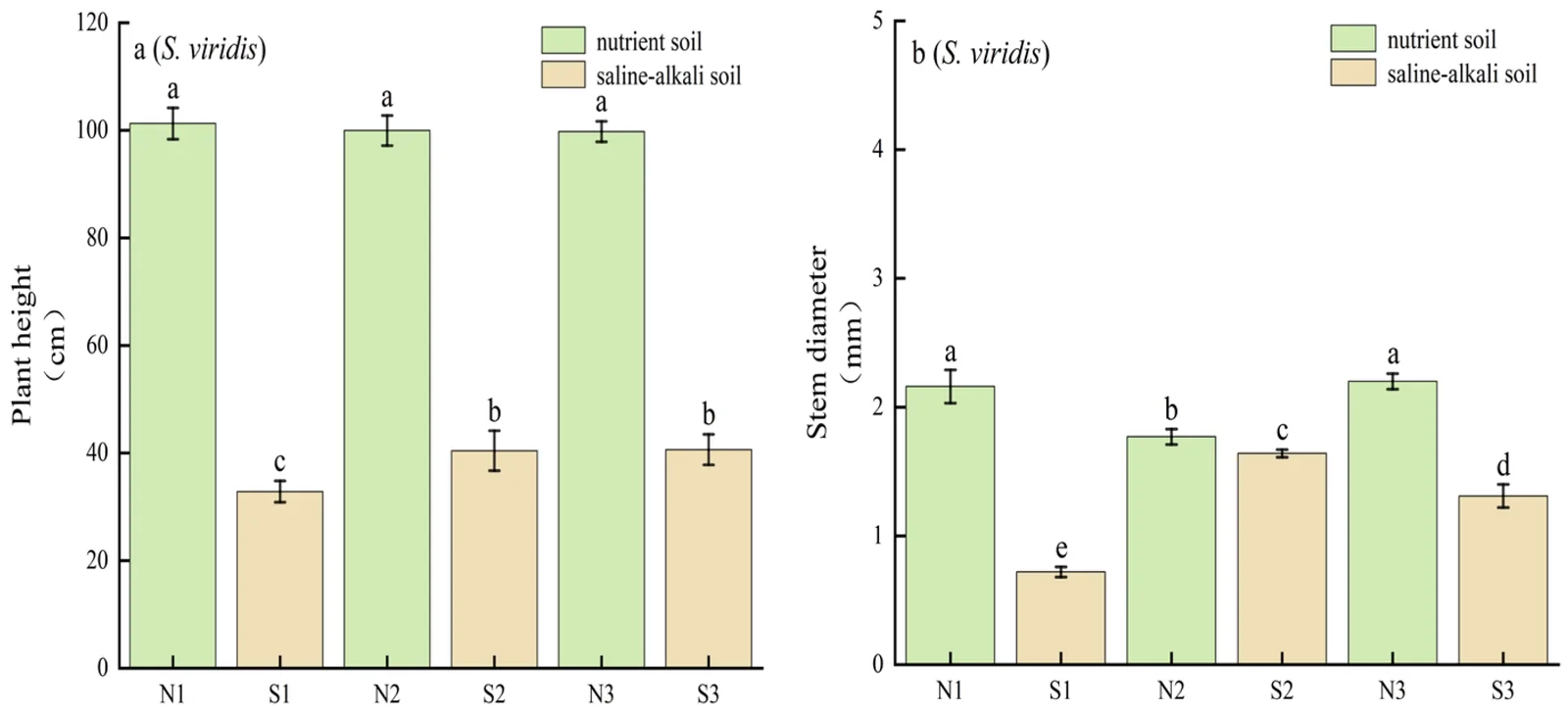

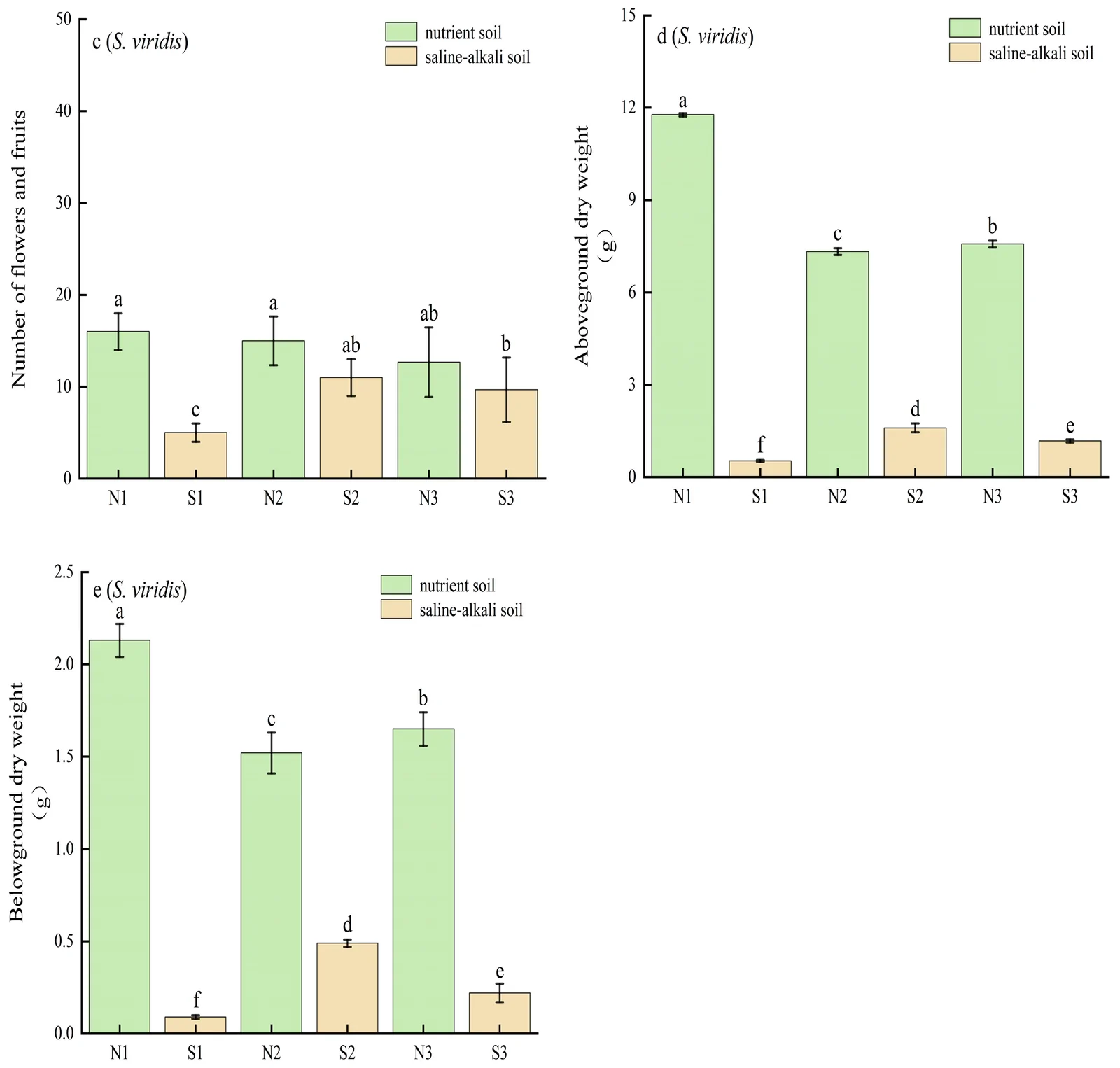
(**a**–**e**) Growth and biomass parameters of *S. viridis.* Note: Different lowercase letters indicate significant differences among plants under different treatments (*p* < 0.05).

In summary, under the nutrient-rich soil substrate, AM fungal inoculation significantly promoted the growth of *S. rostratum*. The growth-promoting effect of the N3 treatment was greater than that of the N2 treatment. In contrast, AM fungal inoculation significantly inhibited stem diameter development and biomass accumulation of *S. viridis* (Figure 4). Under saline-alkali soil conditions, although the overall growth performance of *S. rostratum* was significantly lower than that under nutrient-rich soil conditions, the positive effects of AM fungal inoculation were more pronounced, with the S3 treatment showing a stronger growth-promoting effect than the S2 treatment. For *S. viridis*, all AM fungal inoculation treatments resulted in significantly higher growth performance compared with the non-inoculated control; however, plant growth under the S3 treatment was significantly reduced compared with the S2 treatment, indicating that AM fungal inoculation intensified the competitive suppression of *S. viridis* by *S. rostratum*. These results suggest that AM fungal inoculation, under both nutrient-rich and saline-alkali soil conditions, may enhance the competitive pressure exerted by *S. rostratum* on *S. viridis*, thereby further restricting the growth of the native plant.

### 3.5. Photosynthetic Responses to AM Fungi Inoculation

**Figure 5:**
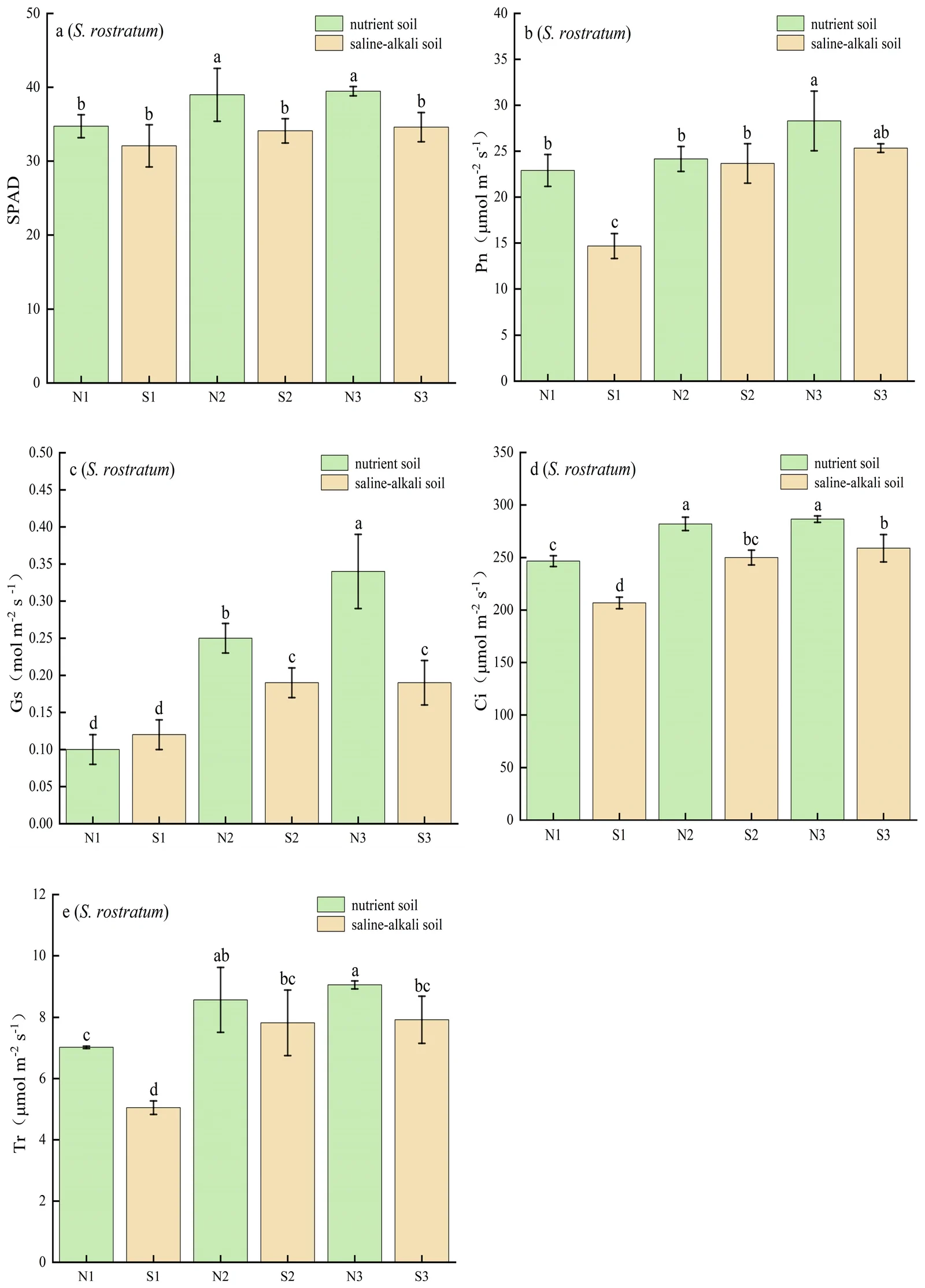
(**a**–**e**) SPAD values and photosynthetic parameters of *S. rostratum.* Note: Different lowercase letters indicate significant differences among plants under different treatments (*p* < 0.05).

**Figure 6:**
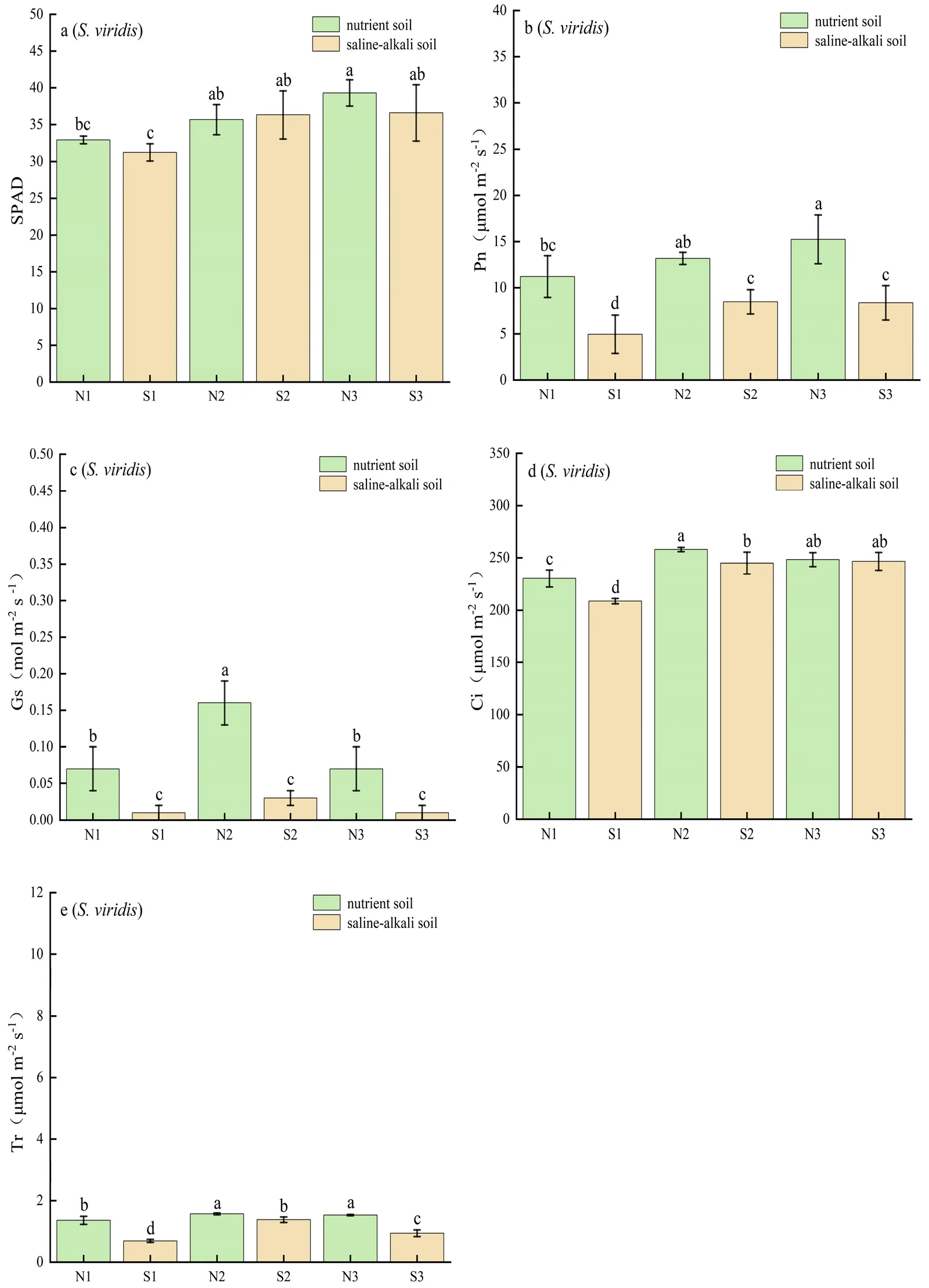
(**a**–**e**) SPAD values and photosynthetic parameters of S. viridis. Note: SPAD values represent the relative chlorophyll content of leaves and are expressed as dimensionless values. Different lowercase letters indicate significant differences among plants under different treatments (*p* < 0.05).

The effects of AM fungal inoculation on SPAD value and photosynthetic parameters of *S. rostratum* and *S. viridis* varied significantly between soil substrates (Figure 5 and Figure 6). Under nutrient-rich soil substrates, AM fungi significantly promoted the SPAD value and photosynthetic parameters of *S. rostratum*. Compared with the non-inoculated treatment N1, treatment N2 exhibited significant increases in SPAD value, stomatal conductance (Gs), intercellular CO_2_ concentration (Ci), and transpiration rate (Tr) (*p* < 0.05), with Ci and Tr increasing by 14.32% and 22.08%, respectively. In treatment N3, SPAD value, net photosynthetic rate (Pn), Gs, Ci, and Tr were all significantly higher than those in treatment N1 (*p* < 0.05). Under saline-alkali soil conditions, photosynthetic performance of *S. rostratum* was reduced; inoculation with AM fungi significantly increased Pn, Gs, Ci, and Tr (*p* < 0.05), with the most pronounced effects observed in treatment S3, where Pn, Gs, Ci, and Tr increased by 72.67%, 58.33%, 25.13%, and 56.83%, respectively. However, no significant effect on SPAD value was detected.

Compared with the invasive plant *S. rostratum*, the responses of SPAD value and photosynthetic parameters of the native plant *S. viridis* to AM fungi were weaker. Under nutrient-rich soil substrates, Ci and Tr in treatments N2 and N3 were significantly higher than those in treatment N1 (*p* < 0.05), but no significant differences in Pn were observed among the three treatments. Moreover, Gs in treatment N3 was significantly lower than that in treatment N2, with a reduction of 56.25% (*p* < 0.05). Similarly, under saline-alkali soil conditions, the photosynthesis of *S. viridis* was also suppressed to some extent. SPAD value, Pn, Ci, and Tr in treatments S2 and S3 were significantly higher than those in treatment S1 (*p* < 0.05), and Gs in treatment S2 was significantly higher than that in treatment S1 (*p* < 0.05). However, Tr in treatment S3 was significantly lower than that in treatment S2, with a reduction of 31.88% (*p* < 0.05).

In summary, the growth-promoting effects of AM fungi on the photosynthetic performance of *S. rostratum* were more pronounced under saline-alkali soil conditions than under nutrient-rich soil conditions. Furthermore, following AM fungal inoculation, most photosynthetic parameters of *S. rostratum* were significantly higher than those of *S. viridis*, indicating that AM fungal symbiosis significantly enhanced the photosynthetic capacity of *S. rostratum*. Consequently, AM fungal inoculation further strengthened the competitive advantage of *S. rostratum* over *S. viridis*.

### 3.6. Effects of AM Fungi on ^15^N Transfer and Allocation Between S. rostratum and S. viridis

AM fungal inoculation under different soil substrates significantly affected ^15^N transfer and allocation in *S. rostratum* and *S. viridis* (Table 6 and Table 7). Under the N1 and S1 treatments without AM fungal inoculation, the δ^15^N values of both *S. rostratum* and *S. viridis* were close to the natural δ^15^N abundance. Under nutrient-rich soil conditions, compared with the N1 treatment, N2 and N3 treatments significantly increased the ^15^N atom% excess in the aboveground parts of *S. rostratum* (*p* < 0.05). Although the ^15^N atom% excess in the aboveground parts of *S. viridis* under N2 and N3 treatments decreased compared with N1, the differences were not significant (*p* > 0.05).

Under saline-alkali soil conditions, the effects of AM fungi on ^15^N acquisition by *S. rostratum* were more pronounced. Compared with the non-inoculated S1 treatment, S2 and S3 treatments significantly increased the ^15^N atom% excess in both aboveground and belowground parts of *S. rostratum* (*p* < 0.05). Notably, although S2 and S3 treatments also significantly increased the ^15^N atom% excess in the aboveground parts of *S. viridis* (*p* < 0.05), the magnitude of increase was lower than that observed in *S. rostratum*. Moreover, under S2 and S3 treatments, the ^15^N atom% excess in the aboveground parts of *S. viridis* was lower than that in *S. rostratum*.

### 3.7. Correlations Among Growth, Photosynthesis and Nutrient Contents

**Figure 7:**
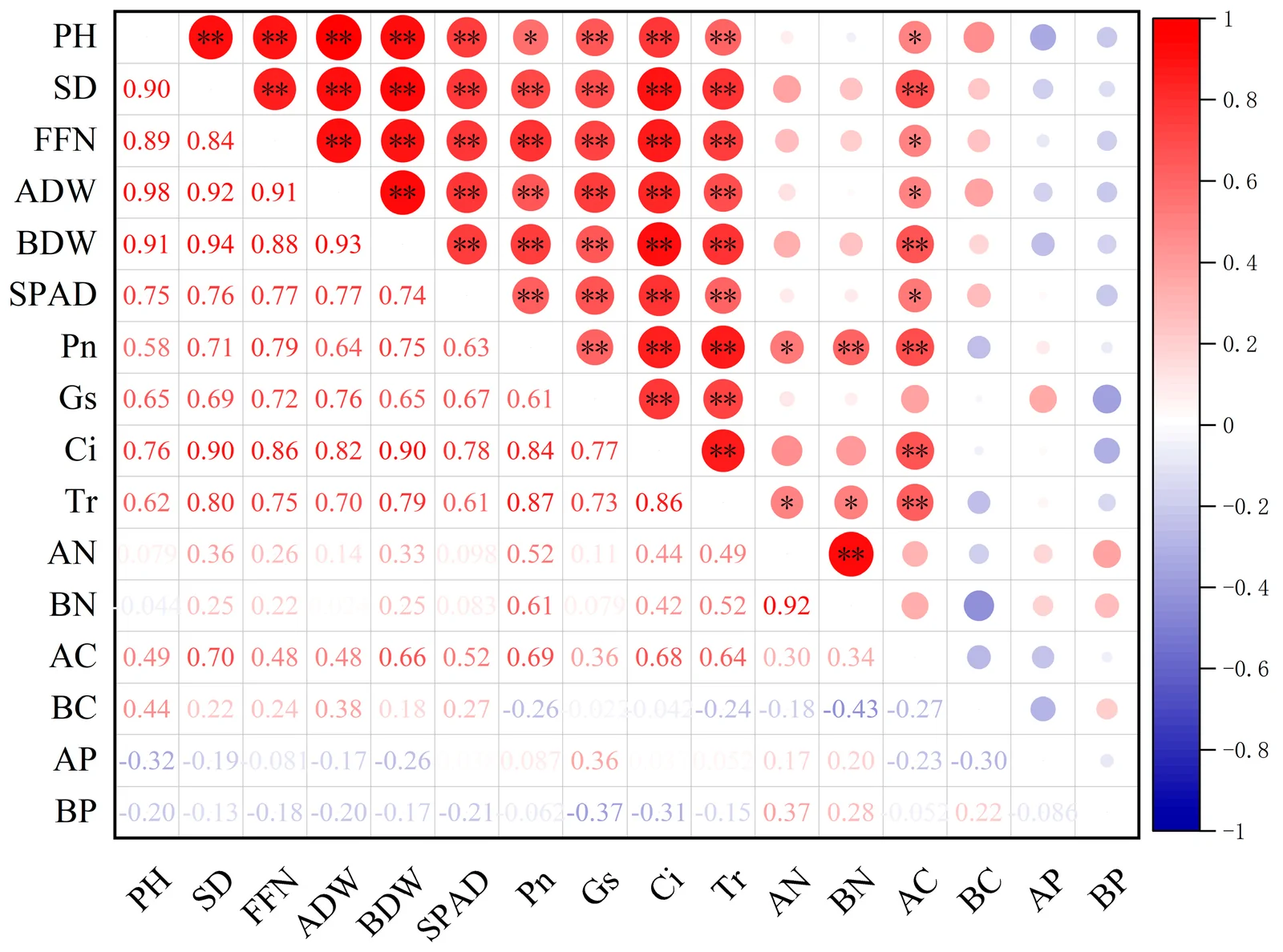
Correlation Analysis among Growth Parameters, Photosynthetic Parameters, and Nutrient Contents in *S. rostratum.* Note: PH: Plant height; SD: Stem diameter; FFN: Number of flowers and fruits; ADW: Aboveground dry weight; BDW: Belowground dry weight; Pn: Net photosynthetic rate; Gs: Stomatal conductance; Ci: Intercellular CO_2_ concentration; Tr: Transpiration rate; AN: Aboveground nitrogen content; BN: Belowground nitrogen content; AC: Aboveground carbon content; BC: Belowground carbon content; AP: Aboveground phosphorus content; BP: Belowground phosphorus content. “*” indicates a significant correlation at the 0.05 level (two-tailed), and “**” indicates a significant correlation at the 0.01 level (two-tailed). In addition, the size of each circle is proportional to the correlation coefficient between the two variables, i.e., larger circles represent stronger correlations.

A Pearson correlation analysis was conducted on the growth indicators, photosynthetic parameters, and carbon (C), nitrogen (N), and phosphorus (*p*) contents in aboveground and belowground parts of *S. rostratum* in the compartmented mesh pot experiment (Figure 7). The results showed that transpiration rate (Tr) was significantly positively correlated with nitrogen contents in both aboveground (AN) and belowground (BN) parts (*p* < 0.05), and was highly significantly positively correlated with aboveground carbon content (AC) (*p* < 0.01). Net photosynthetic rate (Pn) was significantly positively correlated with aboveground nitrogen content (*p* < 0.05), and highly significantly positively correlated with belowground nitrogen content and aboveground carbon content (*p* < 0.01). Additionally, plant height (PH), number of flowers and fruits (FFN), aboveground dry weight (ADW), and SPAD value were all significantly positively correlated with aboveground carbon content (*p* < 0.05); intercellular CO_2_ concentration (Ci), belowground dry weight (BDW), and stem diameter (SD) were highly significantly positively correlated with aboveground carbon content (*p* < 0.01). Notably, significant positive correlations were also observed among the growth indicators and photosynthetic parameters of *S. rostratum* (*p* < 0.05).

### 3.8. Effects of Soil Type, AM Fungi and Their Interaction

The results of the general linear model analysis showed that soil substrate, AM fungi, and their interaction all had significant effects on the growth indicators, photosynthetic parameters, and nutrient element contents in various parts of *S. rostratum* (Table 8). Specifically, soil substrate had significant effects on PH, SD, FFN, ADW, BDW, SPAD, Pn, Gs, Ci, Tr, AC, BC, and AP (*p* < 0.05). AM fungi had a more pronounced effect, significantly influencing all indicators except BP (*p* < 0.05). The interaction between soil substrate and AM fungi also significantly affected PH, ADW, Pn, Gs, AN, BN, AC, BC, and AP of *S. rostratum* (*p* < 0.05).

## 4. Discussion

The role of AM fungi in plant invasion has attracted increasing research attention. However, the effects of AM fungi on invasive plants vary under different soil substrates. Xu et al. [20]. found that AM fungi could still form well-established symbiotic associations with *alfalfa* even under saline-alkali conditions, which is consistent with the results of the present study. Field investigation revealed that AM fungi formed symbiotic structures with *S. rostratum*, with relatively high colonization frequencies ranging from 72.22% to 90.21% and colonization intensities from 13.99% to 29.10%, indicating a strong symbiotic relationship between AM fungi and *S. rostratum*. The high colonization frequency may be a significant factor facilitating the rapid invasion of *S. rostratum*. Majewska et al. [21]. demonstrated that AM fungi promote the growth and phosphorus acquisition of *Solidago canadensis*, further suggesting that AM fungi play a significant role in the rapid invasion of exotic species. Particularly in semi-arid or saline-alkali soil environments, AM fungi enhance the stress tolerance of host plants by improving their nutrient uptake capacity [22]. Previous studies have shown that under different nutrient conditions, AM fungi can increase the nutrient uptake of invasive plants and alter their resource allocation strategies, thereby promoting their growth. By forming mutualistic associations with AM fungi, invasive plants can make full use of limited resources to facilitate their invasion into new environments [12]. Berruti et al. [23]. found that the colonization status of *Camellia* varied among different sampling sites. In the present study, large variations in colonization status were also observed among sampling sites, which may be attributable to differences in soil humus content and soil nutrient availability among the sites [24]. These results indicate that geographic distribution patterns may represent an important factor influencing AM fungal colonization.

The composition of AM fungal communities may vary in the rhizosphere of different plant species or in the rhizosphere of the same plant species under different environmental conditions [25]. In this study, the AM fungal community composition in the rhizosphere of *S. rostratum* varied among different sampling sites, with some AM fungal species appearing only at individual sites, reflecting the uniqueness of AM fungal communities in saline-alkali habitats. This may be because different AM fungi are adapted to different ecological environments, and long-term natural selection helps maintain a stable mycorrhizal system [26]. Furthermore, several studies have shown that species richness of the Glomeraceae family is higher in AM fungal communities in saline soils, with Glomus being the dominant genus [27]. The present study confirmed this finding. High-throughput sequencing revealed that AM fungi in the rhizosphere soil of *S. rostratum* comprised 4 orders, 8 families, 13 genera, and 45 species, among which *Glomus* had the highest relative abundance, with *uncultured Glomus* and *Glomus* sp. being the dominant species. This result is similar to the dominant species identified by Mei et al. [28]. Studies have indicated that *Glomus* exhibits broad adaptability when associating with different hosts under various environmental conditions, mainly due to its high spore production capacity, direct reproduction via hyphae and mycorrhizae [29], and strong resistance to various stress environments, enabling it to thrive under a wide range of conditions [30].

Yang et al. [31]. reported that AM fungi preferentially facilitated the growth of the invasive species *Solidago canadensis*, significantly increasing its biomass and growth indicators after inoculation. In contrast, the growth of co-occurring native plants was not significantly affected by AM fungal inoculation. In the compartmented mesh pot experiment of the present study, AM fungi showed stronger colonization of *S. rostratum* than of *S. viridis*. The colonization frequency, colonization intensity, and arbuscule abundance of *S. rostratum* were significantly higher than those of the native plant *S. viridis*, and this difference was even more pronounced under saline-alkali soil conditions. This difference may enable the invasive plant to gain a growth advantage over the native plant by more efficiently utilizing the mycorrhizal symbiosis [32]. Such efficient utilization may result from the ability of invasive plants to synthesize and release higher concentrations of signaling compounds through their root systems. These compounds may promote AM fungal spore germination and hyphal branching, thereby facilitating the establishment of mycorrhizal associations between invasive plants and AM fungi during the early stages of invasion [33]. Meanwhile, invasive plants generally possess stronger photosynthetic capacity and can allocate more photosynthetic carbon to AM fungi, thereby gaining advantages in carbon–nutrient exchange and acquiring greater amounts of limiting nutrients, such as phosphorus and nitrogen, which may establish positive feedback between invasive plants and AM fungi [34]. Particularly under stressful environments such as saline-alkali soils, the mutualistic benefits between native plants and AM fungi are often constrained, whereas invasive plants can maintain relatively high mycorrhizal responsiveness, further amplifying their growth advantages [35]. This asymmetric preference in symbiotic associations may continuously reshape AM fungal community structure and propagule pools in soils, reduce the availability of mycorrhizal resources for native plants, and consequently intensify competitive exclusion and the spread of invasive species [36]. Furthermore, the present experiment found that *S. rostratum* competes for AM fungal resources in the soil. In the treatment where AM fungi were inoculated only into the root compartment of the native plant *S. viridis*, *S. rostratum* was still colonized by AM fungi. This may also result from the allelopathic compounds synthesized and released by the roots of *S. rostratum*.

Chen et al. [37]. found that nitrogen can be transferred between invasive and native plants via AM fungal hyphal connections. In the compartmented mesh pot experiment, AM fungi and the stable isotope ^15^N were added only to the *S. viridis* compartment; nevertheless, *S. rostratum* in the adjacent compartment was also colonized by AM fungi, and a significant increase in ^15^N atom% excess content was detected in its tissues. Notably, under saline-alkali soil conditions, the increase in aboveground ^15^N atom% excess in *S. rostratum* was substantially greater than that in *S. viridis*, indicating that a common mycorrhizal network had formed between the two compartments. This mycelial network can connect the roots of the same or different plant species and facilitate the transfer of nutrients among individual plants [38]. Such nitrogen transfer is likely to be asymmetric. Invasive plants, with their stronger carbon assimilation capacity, may provide sufficient photosynthetic carbon to the hyphal network, thereby promoting the preferential transfer of nitrogen by AM fungi toward the roots of invasive plants [39]. This asymmetry may be further amplified under saline-alkali stress, where carbon supply from native plants is constrained, resulting in a greater allocation of nitrogen through the hyphal network toward invasive plants and creating a nutrient transfer imbalance that favors invasive species. In this experiment, the belowground common mycorrhizal network formed between the invasive plant and the native plant enabled *S. rostratum* to compete for AM fungal resources and nutrients such as nitrogen in the soil surrounding *S. viridis*, thereby giving *S. rostratum* a competitive advantage over *S. viridis* [40]. Correlation analysis results showed that the growth and photosynthetic parameters of *S. rostratum* were significantly positively correlated with its internal nutrient contents, and growth parameters were also significantly positively correlated with photosynthetic parameters. Thus, when *S. rostratum* acquires more key nutrient resources than the native plant, its photosynthetic parameters—such as net photosynthetic rate and stomatal conductance—are correspondingly and significantly enhanced compared with those of *S. viridis* [41], thereby improving nutrient conversion efficiency in *S. rostratum* and further promoting its growth [42].

In addition to mediating nutrient transport via mycelial networks, environmental factors such as soil salinization critically modulate host-fungal dynamics. The most direct effect of saline-alkali stress on plants is growth inhibition, manifested as a comprehensive decline in key indicators such as total biomass, plant height, leaf area, and root development [43]. The general linear model analysis showed that soil type was a significant factor affecting the growth of *S. rostratum*, with saline-alkali soil significantly suppressing its growth. In our experiment, all growth indicators of plants under saline-alkali soil conditions were significantly lower than those under nutrient-rich soil conditions. This is mainly because plants grown in saline-alkali soils are subjected to osmotic stress and ion toxicity, which severely restrict root water uptake, photosynthesis, and nutrient acquisition, thereby inhibiting plant growth [44]. The salt-alkali tolerance of plants is generally positively correlated with their ability to maintain biomass under stress; therefore, changes in biomass are a key indicator for evaluating stress tolerance [45]. Similarly, substrate-dependent responses to AM fungal inoculation have been observed in other plant systems; for instance, Moraes et al. [46]. demonstrated that the effects of AM fungi on own-rooted ‘Koroneiki’ olive nursery trees varied significantly between different soil substrates. The compartmented mesh pot experiment in this study revealed that, under both saline-alkali and nutrient-rich soil substrates, AM fungi significantly affected various physiological and ecological indicators of *S. rostratum*, and this difference was even more pronounced under saline-alkali conditions. This indicates that the symbiotic effect between AM fungi and plants is highly dependent on environmental conditions, and that AM fungi may play a more critical ecological regulatory role under stress conditions [47]. This may be mainly because saline-alkali stress restricts the ability of plant roots to independently acquire water and nutrients [44]. AM fungi can compensate for these functional limitations by extending extraradical hyphae to enlarge the absorption area and alleviate ion toxicity, thereby significantly improving plant performance under saline-alkali conditions [48]. Meanwhile, under stress conditions, plants may obtain greater benefits from exchanging equivalent amounts of photosynthetic carbon for mycorrhizal-derived nutrients and water, resulting in increased mycorrhizal dependency. Consequently, the ecological regulatory functions of AM fungi may be further amplified under stressful environments. Saline-alkali stress itself significantly inhibited the growth of *S. rostratum*, but inoculation with AM fungi largely alleviated this inhibition, and the alleviation effect was more comprehensive and efficient in *S. rostratum*. Meanwhile, under saline-alkali conditions, AM fungi further enhanced the competitive effect of *S. rostratum* on *S. viridis*. This may be because the available nutrient content in saline-alkali soil is significantly lower than that in nutrient-rich soil, leading to more intense resource competition among plants [49]. In this stress environment, *S. rostratum*, through its efficient symbiotic system formed with AM fungi, can not only make fuller use of limited soil nutrients but also indirectly acquire nutrient resources from the rhizosphere of *S. viridis* via the hyphal network, thereby strengthening its resource competitive advantage [50].

## 5. Conclusions

In the saline-alkali region of western Jilin Province, the invasive plant *S. rostratum* forms a stable symbiotic association with AM fungi, with *Glomus* being the dominant AM fungal genus in its rhizosphere. The effects of AM fungi on *S. rostratum* vary significantly between soil substrates, with more pronounced effects observed under the saline-alkali soil substrate. In interspecific competition, AM fungi showed a clear preference for *S. rostratum*, with significantly higher colonization frequency, colonization intensity, and arbuscule abundance than those in the native plant *S. viridis*, particularly under saline-alkali soil conditions. Moreover, *S. rostratum* not only competed for AM fungal resources associated with *S. viridis* but also acquired nutrients from *S. viridis* through the common mycorrhizal network, as demonstrated by the ^15^N compartment experiment, indicating that hyphal networks facilitated nitrogen transfer between plants and enhanced the competitive advantage of *S. rostratum*.

AM fungal inoculation significantly increased biomass accumulation, net photosynthetic rate, and nitrogen acquisition efficiency of *S. rostratum*, with stronger effects observed in saline-alkali soil than in nutrient-rich soil, and these responses were greater than those observed in *S. viridis* under the same soil conditions. Overall, soil type was a key factor influencing *S. rostratum* growth, with saline-alkali soil suppressing plant performance, whereas AM fungal inoculation effectively alleviated these negative effects, especially in *S. rostratum*. Under saline-alkali stress, AM fungi enhanced nutrient acquisition and photosynthetic performance of *S. rostratum*, improving its stress tolerance and competitive ability, suggesting that AM fungi play an important role in the adaptation and invasion success of *S. rostratum*.

Understanding AM fungal-mediated interactions between invasive and native plants provides insights into invasion mechanisms and offers a theoretical basis for managing invasive species by regulating plant–AM fungal symbioses. Future studies should further investigate the role of AM fungi in nutrient competition and exchange networks between invasive and native plants, which will improve predictions of mycorrhiza-mediated invasion dynamics and ecosystem responses related to nutrient allocation and community stability.

## Figures and Tables

**Table 1:** Experimental design and treatments.

Treatment	Soil Substrate	Chamber I	Chamber II
N1	nutrient-rich soil substrate	*S*. *viridis* (+^15^N)	*S. rostratum*
N2	nutrient-rich soil substrate	*S*. *viridis* + AM fungi (+^15^N)	*S. rostratum*
N3	nutrient-rich soil substrate	*S*. *viridis* + AM fungi (+^15^N)	*S. rostratum* + AM fungi
S1	saline-alkali soil substrate	*S*. *viridis* (+^15^N)	*S. rostratum*
S2	saline-alkali soil substrate	*S*. *viridis* + AM fungi (+^15^N)	*S. rostratum*
S3	saline-alkali soil substrate	*S*. *viridis* + AM fungi (+^15^N)	*S. rostratum* + AM fungi

**Table 2:** Colonization Status and Spore Density of *S. rostratum*.

Sampling Site	Colonization Frequency	Colonization Intensity	Arbuscule Abundance	Spore Density
	F (%)	M (%)	a (%)	(Spores/100g)
XZC	72.22 ± 11.70 b	15.96 ± 5.6 cd	8.83 ± 3.04 ab	286.00 ± 7.94 a
LJC	86.67 ± 8.82 a	27.22 ± 5.89 ab	13.27 ± 4.54 ab	78.67 ± 5.77 g
LJY	84.44 ± 5.09 ab	24.99 ± 11.05 ab	13.68 ± 3.06 ab	116.67 ± 7.37 e
CSC	83.33 ± 12.02 ab	23.27 ± 13.98 abc	11.08 ± 6.74 ab	103.33 ± 3.056 f
JCZ	76.67 ± 3.34 ab	13.99 ± 5.75 d	9.40 ± 3.43 ab	159.67 ± 4.73 d
BDG	83.33 ± 6.67 ab	20.15 ± 1.59 cd	11.96 ± 1.93 ab	244.00 ± 11.27 b
LYT	81.11 ± 1.92 ab	29.10 ± 5.16 a	19.65 ± 6.09 a	119.00 ± 7.21 e
EQS	90.21 ± 8.45 a	28.88 ± 10.35 a	17.30 ± 5.63 a	216.33 ± 9.50 c
YST	80.01 ± 3.35 ab	14.33 ± 9.69 d	8.36 ± 8.17 b	81.33 ± 9.02 g
YLH	85.55 ± 6.94 ab	22.46 ± 10.12 bcd	17.87 ± 7.42 a	61.33 ± 5.77 h

Note: Different lowercase letters indicate significant differences among sampling sites (*p* < 0.05).

**Table 3:** Species Composition of AM fungi in the Rhizosphere of *S. rostratum*.

Order	Family	Genus	Species
Archaeosporales	Archaeosporaceae	*Archaeospora*	*Archaeospora* sp.
		(<1%)	
	Ambisporaceae	*Ambispora*	*Ambispora* sp.
		(<1%)	
			*Ambispora fennica*
Diversisporales	Acaulosporaceae	*Acaulospora*	*Acaulospora* sp.
		(<1%)	
	Diversisporaceae	*Diversispora*	*Diversispora* sp.

		(2.85%)	*Diversispora spurca*
			*Diversispora aurantia*
	Gigasporaceae	*Scutellospora*	*Scutellospora aurigloba*
		(<1%)	
			*Scutellospora calospora*
			*Scutellospora* sp.
Glomerales	Claroideoglomeraceae	*Claroideoglomus* (22.37%)	*Claroideoglomus claroideum*
			*Claroideoglomus etunicatum*
			*Claroideoglomus lamellosum*
			*Claroideoglomus* sp.
			*Claroideoglomus* sp._*torrecillas*
	Glomeraceae	*Dominikia*	*Dominikia* sp.
		(<1%)	
		*Funneliformis*	*Funneliformis* sp.
		(2.50%)	*Funneliformis mosseae*
		*Glomus*	*Glomus indicum*
		(57.50%)	
			*Glomus* sp.

			*Glomus* sp. *0715-3*
			*Glomus* sp. *8 FW-2016*
			*Glomus* sp. *C/2-2*
			*Glomus* sp. *C/2-3*
			*Glomus* sp. *G06*
			*Glomus* sp. *Glo39*
			*Glomus* sp. *Glo56*
			*Glomus* sp. *goomaral*
			*Glomus* sp. *MC27*
			*Glomus* sp. *NBR PP1*
			*Glomus* sp. *OGS11*
			*Glomus* sp. *OGS12*
			*Glomus* sp. *VeGlo8*
			*Glomus* sp. *VeGloB*
			*Glomus* sp. *W3234*
			*Glomus* sp. *yamato 09*
		*Rhizoglomus*	*Rhizoglomus* sp.
		(<1%)	
		*Rhizophagus*	*Rhizophagus intraradices*
		(9.75%)	*Rhizophagus iranicus*
			*Rhizophagus irregularis*
			*Rhizophagus* sp. *(in: Fungi)*
		*Septoglomus*	*Septoglomus constrictum*
		(<1%)	
			*Septoglomus* sp.
			*Septoglomus viscosum*
Paraglomerales	Paraglomeraceae	*Paraglomus*	*Paraglomus* sp.
		(<1%)	

**Table 4:** Symbiotic status of AM fungi in *S. rostratum*.

Treatments	Plant	Colonization Frequency	Colonization Intensity	Arbuscule Abundance
		F (%)	M (%)	a (%)
N1	S. rostratum	0	0	0
N2	S. rostratum	61.84 ± 1.41 c	20.52 ± 0.50 c	3.26 ± 0.61 b
N3	S. rostratum	76.67 ± 3.34 a	28.61 ± 2.07 ab	9.07 ± 0.72 a
S1	S. rostratum	0	0	0
S2	S. rostratum	47.78 ± 3.85 d	7.11 ± 1.05 d	0.19 ± 0.04 c
S3	S. rostratum	71.11 ± 1.92 b	32.79 ± 5.21 a	9.82 ± 0.45 a

**Table 5:** Symbiotic status of AM fungi in *S. viridis*.

Treatments	Plant	Colonization Frequency	Colonization Intensity	Arbuscule Abundance
		F (%)	M (%)	a (%)
N1	*S. viridis*	0	0	0
N2	*S. viridis*	66.67 ± 3.34 a	14.91 ± 1.00 a	0.46 ± 0.06 c
N3	*S. viridis*	68.51 ± 2.59 a	14.96 ± 2.54 a	1.85 ± 0.14 a
S1	*S. viridis*	0	0	0
S2	*S. viridis*	44.45 ± 3.85 b	8.31 ± 0.88 b	0.35 ± 0.04 c
S3	*S. viridis*	65.14 ± 2.66 a	16.46 ± 3.58 a	0.85 ± 0.15 b

Note: N1, N2, and N3 indicate no AM fungal inoculation, AM fungal inoculation only in *Setaria viridis*, and AM fungal inoculation in both *Solanum rostratum* and *Setaria viridis* under nutrient-rich soil conditions, respectively. S1, S2, and S3 indicate the corresponding treatments under saline-alkali soil conditions. Different lowercase letters indicate significant differences among treatments within each plant species (*p* < 0.05).

**Table 6:** Transport and Allocation of ^15^N in *S. rostratum* under Different Treatments.

Treatments	Plant	Aboveground ^15^N	Belowground ^15^N
		Abundance	Abundance
N1	*S. rostratum*	0.39 ± 0.01 d	0.39 ± 0.01 b
N2	*S. rostratum*	0.49 ± 0.01 c	0.38 ± 0.01 b
N3	*S. rostratum*	0.55 ± 0.07 b	0.41 ± 0.02 b
S1	*S. rostratum*	0.37 ± 0.01 d	0.38 ± 0.01 b
S2	*S. rostratum*	0.58 ± 0.02 b	0.49 ± 0.07 a
S3	*S. rostratum*	0.64 ± 0.03 a	0.42 ± 0.03 ab

**Table 7:** Transport and Allocation of ^15^N in *S. viridis* under Different Treatments.

Treatments	Plant	Aboveground ^15^N	Belowground ^15^N
		Abundance	Abundance
N1	*S. viridis*	0.86 ± 0.18 a	0.44 ± 0.05
N2	*S. viridis*	0.83 ± 0.09 a	0.47 ± 0.08
N3	*S. viridis*	0.77 ± 0.08 a	0.46 ± 0.08
S1	*S. viridis*	0.42 ± 0.07 c	0.41 ± 0.04
S2	*S. viridis*	0.52 ± 0.02 b	0.45 ± 0.03
S3	*S. viridis*	0.59 ± 0.09 b	0.52 ± 0.08

Note: N1, N2, and N3 represent the treatments in nutrient-rich soil without AM fungal inoculation, with AM fungal inoculation of *Setaria viridis* only, and with AM fungal inoculation of both *S. rostratum* and *S. viridis*, respectively. S1, S2, and S3 represent the treatments in saline-alkali soil without AM fungal inoculation, with AM fungal inoculation of *S. viridis* only, and with AM fungal inoculation of both *S. rostratum* and *S. viridis*, respectively. In this experiment, the ^15^N isotope was applied exclusively to the *S. viridis* compartment. Different letters denote significant differences among treatments within each plant species (*p* < 0.05).

**Table 8:** Effects of Soil Substrate, AM fungi, and Their Interaction on *S. rostratum*.

	Soil	AM Fungi	Soil × AM Fungi
PH	558.75 *	29.19 *	7.82 *
SD	240.54 *	51.96 *	3.03
FFN	62.29 *	18.51 *	0.61
ADW	1404.33 *	176.19 *	42.51 *
BDW	280.21 *	46.16 *	3.75
SPAD	15.22 *	4.60 *	0.48
Pn	18.46 *	26.84 *	6.37 *
Gs	22.41 *	46.01 *	15.15 *
Ci	91.50 *	68.13 *	1.02
Tr	15.36 *	22.17 *	1.21
AN	0.32	18.19 *	30.39 *
BN	0.4	11.56 *	17.08 *
AC	12.27 *	11.7 *	6.47 *
BC	14.76 *	5.18 *	8.76 *
AP	45.95 *	29.92 *	8.62 *
BP	0.07	2.11	1.26

Note: “*” indicates significance at *p* < 0.05 (two-tailed).

## Data Availability

The original contributions presented in this study are included in the article. Further inquiries can be directed to the corresponding authors.
